# Beneficial Effects of Xanthohumol on Metabolic Syndrome: Evidence from In Vitro and Animal Model Studies

**DOI:** 10.3390/ijms252212434

**Published:** 2024-11-19

**Authors:** Saioa Gómez-Zorita, Carina Proença, Alfredo Fernández-Quintela, Isabel Moreno-Indias, María P. Portillo

**Affiliations:** 1 Nutrition and Obesity Group, Department of Nutrition and Food Science, Faculty of Pharmacy, University of the Basque Country (UPV/EHU), 01006 Vitoria-Gasteiz, Spain; alfredo.fernandez@ehu.eus (A.F.-Q.); mariapuy.portillo@ehu.eus (M.P.P.); 2 Lucio Lascaray Research Center, 01006 Vitoria-Gasteiz, Spain; 3 CIBERobn Physiopathology of Obesity and Nutrition, Institute of Health Carlos III (ISCIII), 28029 Madrid, Spain; isabel.moreno@ibima.eu; 4 BIOARABA Health Research Institute, 01006 Vitoria-Gasteiz, Spain; 5 LAQV, REQUIMTE, Laboratory of Applied Chemistry, Department of Chemical Sciences, Faculty of Pharmacy, University of Porto, 4050-313 Porto, Portugal; cproenca@ff.up.pt; 6 Department of Endocrinology and Nutrition, Virgen de la Victoria Hospital (IBIMA), Malaga University, 29590 Malaga, Spain

**Keywords:** xanthohumol, beer, metabolic syndrome, in vitro, animal models

## Abstract

Metabolic syndrome refers to the simultaneous occurrence of several disorders that have been associated with other co-morbidities, such as a pro-inflammatory state and non-alcoholic fatty liver disease. Nowadays, it is a growing public health problem that contributes to the development of non-communicable diseases, such as type 2 diabetes, cardiovascular disease, and cognitive deficits among others. Its incidence has been related to modifiable lifestyle factors, mainly dietary patterns and physical activity. In addition, numerous studies have observed the potential beneficial effects of polyphenols in the prevention and treatment of metabolic syndrome components in both animals and humans. In this line, the aim of this review is to present the scientific evidence available about the beneficial effects of the phenolic compound xanthohumol in the prevention and/or treatment of obesity, dyslipidemia, insulin resistance, and fatty liver, which are important components of metabolic syndrome. All the potential beneficial effects described in this manuscript have been observed in vitro and in animal models, there are no published clinical trials in this context yet.

## 1. Introduction

Metabolic syndrome refers to the simultaneous occurrence of several disorders including insulin resistance, (central) obesity, dyslipidemia, and hypertension. It has been associated with other co-morbidities, such as a pro-inflammatory state and metabolic dysfunction-associated steatotic liver disease (MASLD). Multiple organizations, like the World Health Organization (WHO) and the International Diabetes Federation, have their own definition for this syndrome. These definitions includes all the mentioned disorders, but the cut-offs of these alterations vary among them [1,2]. Metabolic syndrome is a growing public health problem that contributes to the development of non-communicable diseases, such as type 2 diabetes, cardiovascular disease, and cognitive deficits, among others.

Scientific evidence has demonstrated the association between the incidence of metabolic syndrome and modifiable lifestyle factors, mainly dietary patterns and physical activity [3]. In addition, numerous studies have highlighted the potential beneficial effects of polyphenols in the prevention and treatment of metabolic syndrome components in both animals and humans [1,4,5].

The aim of the present narrative review is to present the scientific evidence available on the beneficial effects of the phenolic compound xanthohumol in the prevention and/or treatment of several components of metabolic syndrome, obesity, dyslipidemia, insulin resistance, and fatty liver. To date, reported studies have been conducted either in culture pre-adipocytes, mature adipocytes and hepatocytes, or in animal models, but the studies addressed in human beings are lacking.

## 2. Xanthohumol

**Figure 1:**
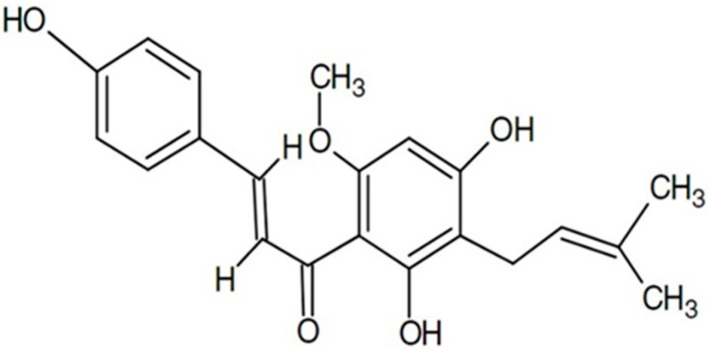
Chemical structure of xanthohumol.

Xanthohumol is a phenolic compound belonging to the group of flavonoids, more specifically to prenylflavonoids, which presents a structure of chalcone (Figure 1). It is found in the female inflorescences of *Humulus lupulus* (Cannabaceae), also known as hops (0.1–1 g/100 g dry weight) [6,7]. This compound belongs to a class of compounds that contribute to the bitterness and flavor of hops [8].

Bioavailability of bioactive compounds is an important issue to be studied when the potential use of bioactive compounds to prevent or to treat diseases is analyzed. Avula et al. (2004) found only 0.06% to 0.49% of the administered xanthohumol in the urine and more than 99.5% in the faeces of rats, suggesting that this compound is poorly absorbed through the intestinal wall [9]. In line with these results, in other studies, after oral administration of xanthohumol to rats, most of the phenolic compound (89%) appeared unchanged in the faeces, which is indicative of its poor bioavailability [10,11].

Phenolic compounds are metabolised in the gut and liver by phase II metabolic enzymes. It is well known that glucuronides are the main phase II metabolites of flavonoids. In the case of phenylated flavonoids, such as xanthohumol, the first data concerning this issue were provided by Yilmazer et al. (2001) [12]. The authors incubated isolated human and rat liver microsomes with xanthohumol and observed that four glucuronide metabolites were produced, two of them represented 89% and 10% of total glucuronides formed in incubations, whereas the other two metabolites only accounted for about 1% of total glucuronides. Later on, Ruefer et al. (2005) proposed that both glucuronidation and sulfation could participate in phase II metabolism of xanthohumol in the liver, as well as in the gastrointestinal tract [13].

Animal model studies have also been addressed. Thus, after oral administration of xanthohumol (50 mg/kg body weight) to male rats, the parent phenolic compound appeared in plasma mainly in the form of two mono-glucuronides which reached maximum concentrations of 180 and 65 nM, respectively, 4 h after the administration. In a study reported by Legette et al., plasma total xanthohumol (sum of free compound and conjugated metabolites) recovery was 33%, 13%, and 11%, respectively, over 96 h after oral administration of xanthohumol at doses of 1.86, 5.64, and 16.9 mg/kg body weight [14]. Moreover, another study where higher doses were used (40, 100, and 200 mg xanthohumol/kg body weight) confirmed the absolute bioavailability was 1.16%, 0.96%, and 0.53%, respectively [15].

All together, these data show that, as in the case of the vast majority of phenolic compounds, xanthohumol presents a low bioavailability. The amounts of these glucuronides excreted in the urine accounted for 0.3% and 0.05% of the administered dose, respectively, which indicated that alternative routes of elimination, such as biliary secretion, might be more prominent. This fact has also been recently confirmed by Bai et al. (2022) [16]. Although data concerning the bioavailability of xanthohumol in humans are scarce, they suggest that the pharmacokinetics of xanthohumol in metabolically healthy humans and animals is similar [17]. Thus, the administration of hop extract capsules containing 21, 43, or 85 mg xanthohumol in post-menopausal women led to maximum concentrations of 4.4 ng/mL, 22.2 ng/mL, and 27.6 ng/mL. These concentrations were achieved after 3.8 h, 3.6 h, and 1.8 h, respectively. [18]. The authors explain that these data may seem surprising, saying that, at the highest dosages, uptake and storage of these compounds in the colon epithelium (that can act as a reservoir for xanthohumol release into circulation) might become saturated, thereby justifying the shorter time to serum Tmax at high doses.

Potential toxicity is another relevant aspect in the study of bioactive compounds. In the work addressed by Dorn et al. (2010), the administration of 1000 mg of xanthohumol/kg body weight/day to female mice for 3 weeks did not reveal any sign of toxicity, based on histopathological examination of different tissue and biochemical serum analysis [19]. Weiskirchen et al. (2015) confirmed the safety of xanthohumol by administering 700 or 1000 mg/kg body weight/day to mice for 4 and 3 weeks, respectively, and showed no negative effects [20]. As far as human studies are concerned, different studies have confirmed the safety of this phenolic compound in healthy individuals [21], including menopausal women [18].

The content of xanthohumol in hops is high, but its concentration in beer is much lower because the thermal treatment used in the production process of this drink induces the isomerization of xanthohumol to isoxanthohumol. Moreover, other processes involved in beer production, such as fermentation and filtration, also reduce the xanthohumol content. As a consequence, the amount of xanthohumol found in beers ranges from 0.002 to 0.690 mg/L, whereas that of isoxanthohumol ranges from 0.040 to 0.716 mg/L [17,22]. Considering the interest of xanthohumol due to its potential beneficial effects on health, variations of the brewing process have been developed to increase xanthohumol content in beer of up to 20 mg/L [17].

## 3. In Vitro Studies

To date, several in vitro studies have been conducted to analyze the effects of xanthohumol mainly in murine adipocytes (Table 1), but also in hepatocytes. Yang et al. (2007) carried out a study in 3T3-L1 pre-adipocytes by adding the phenolic compound at 3.125, 6.25, 12.5, or 25 μM to the culture medium, during the 6 days of the differentiation period [23]. All the doses significantly reduced triglyceride accumulation in a dose-dependent manner, without affecting cell viability. Regarding the anti-adipogenic mechanism, peroxisome proliferator-activated receptor γ (PPARγ), CCAAT/enhancer binding protein α (C/EBPα), fatty acid-binding protein 2 (aP2), and diacylglycerol O-acyltransferase 1 (DGAT1) protein expression were decreased by the treatment.

In the same study, mature 3T3-L1 adipocytes (day 8 of differentiation period) were also incubated during 24 or 48 h with xanthohumol at 25, 50, 75, or 100 μM. From 50 to 100 μM, this compound significantly reduced cell viability, suggesting this effect was more prominent with the longest incubation period (48 h), and increased cell apoptosis, suggesting that it was implicated in the reduction of cell viability. All the following determinations were carried out only at 75 μM. To analyze whether oxidative stress was implicated in the increased apoptosis, reactive oxygen species (ROS) production was measured. In this sense, an increase in ROS production was observed when xanthohumol was added to the adipocytes. In addition, the phenolic compound significantly reduced mitochondrial membrane potential, and this reduction was associated with the release of cytochrome c from mitochondria, as it was observed by the increase in cytoplasmic cytochrome c protein expression. In view of these results, the authors proposed that ROS production induced apoptosis via a mitochondria-dependent pathway. Moreover, caspase 3/7 activation was also increased, an effect probably induced by cytochrome c. To confirm caspase 3 activation, cleaved poly (ADP-ribose) polymerase (PARP) protein expression was determined. As expected, full-length protein disappeared, and PARP fragments were accumulated. In summary, xanthohumol inhibited adipogenesis, and enhanced apoptosis by an increase in intracellular ROS production.

Later on, the same authors carried out a similar experiment, but in this case with lower doses of the compound [24]. The 3T3-L1 pre-adipocytes were treated with xanthohumol at 1.5 or 3.12 μM during the 6 days of the differentiation period. Both doses significantly reduced lipid accumulation, in a dose-response manner, without decreasing cell viability. When they studied the effects on the transcription factor involved in adipogenesis regulation, no differences in PPARγ, C/EBPα, aP2, or DGAT1 protein expression were observed. No further explanation was provided to justify the anti-adipogenic effect.

In addition, mature adiposities, on day 6 after the differentiation period, were incubated with the phenolic compound at 12.5 or 25 μM for 24 or 48 h. The highest dose of xanthohumol reduced cell viability. Nevertheless, it did not significantly increase adipocyte apoptosis, although caspase 3/7 activity, measured after 24 h of treatment, was increased by the highest dose. Other determinations were only carried out with the highest dose (25 μM). No differences in the critical regulators of the apoptotic pathways B cell Lymphoma (BCL2) and (Bcl-2)-associated X (BAX) protein expression were observed between control and treated cells. With regard to lipid metabolism, the authors observed an increase in lipolysis, measured as glycerol release.

Mendes et al. (2008) studied the effect of xanthohumol (from 0.01 to 20 µM) in different periods of cellular differentiation, in order to identify in which phase of the adipogenic differentiation this compound was most relevant, and thus to better characterize its mechanism of action [25]. Xanthohumol was added (a) from the day of confluence (day −2) to day 7 of differentiation (from day −2 to day 7), (b) from the day of confluence to the start of differentiation (day 0) (from day −2 to day 0), (c) during the 7 days of differentiation period (from day 0 to day 7), (d) during the first 2 days of the differentiation (from day 0 to day 2), or (e) during the last 5 days of the differentiation process (from day 2 to day 7).

Xanthohumol reduced lipid accumulation most strongly when cells were incubated with the compound 24 h after plating, until full differentiation (a); but, on the contrary, it had little or no effect when added to the culture medium after the adipogenic challenge (e). Regarding adipogenesis, the doses of 0.01 or 0.1 μM were ineffective. At 10 and 20 µM, adipogenesis was reduced in all periods, with the exception of the treatment from day 2 to day 7 (e); and at 1 µM it was also decreased, with the exception of the periods from day 2 to day 7 (e), and from day −2 to day 0 (b). Due to the fact that in adipogenesis, two different processes, cell proliferation and cell differentiation, can be distinguished, the authors analyzed the effect of xanthohumol for each process.

When the effects on cell proliferation were assessed, the authors observed that xanthohumol reduced protein content in a dose-dependent manner. The half-maximal inhibitory concentration (IC50) for cell growth were 12–26 µM, which were in the range of concentration that reduced adipogenesis. Moreover, when the treatment was carried out for 48 or 72 h, the number of pre-adipocytes was lower than at the beginning of the treatment, suggesting not only an inhibition of cell growth but a cytotoxic effect. In view of this result, the effect of 5 µM of the phenolic compound for 24 h on apoptosis was evaluated, thus confirming that xanthohumol significantly increased apoptosis, as it was observed by nuclear staining. Additionally, the expression of the proliferation-specific nuclear protein Ki67 was measured when pre-adipocytes were incubated with xanthohumol at 1 or 5 µM for 24 h. At 5 µM a decrease in this protein content was observed, indicating a good agreement with the determination in protein content, and a lower number of cells. Altogether, these results demonstrated that this polyphenol was able to reduce cell proliferation.

Taking into account that PPARγ is the master regulator of adipogenesis, the authors studied whether this transcriptional factor was involved in the anti-adipogenic effect of xanthohumol, by analyzing its protein expression when the phenolic compound was added at 10 µM to the cells from day 0 of the differentiation period to day 7 (c). A reduction in the expression of PPARγ was observed. Moreover, NFκB, a nuclear transcription factor involved in the regulation of different genes related to apoptosis and cell proliferation was also measured. The treatment with 10 µM of xanthohumol for 24 h after seeding slightly increased NFκB activity. By contrast, 10 µM of xanthohumol for 24 h on day 7 did not modify NFκB activity.

Samuels et al. (2018) analyzed whether xanthohumol (6.25 or 25 µM, doses that are not cytotoxic in these cells) was able to reduce adipogenesis in 3T3-L1 adipocytes and human subcutaneous adipocytes [26]. At the end of the 6 days of the differentiation period, the authors observed a decrease in lipid content, indicating an inhibitory effect of the phenolic compound.

On mature adipocytes, these same authors investigated the anti-obesity effects of xanthohumol, and particularly its effects on the browning process, as well as other metabolic pathways involved in the delipidating effect. For this purpose, 3T3-L1 adipocytes were treated with xanthohumol 6.25 or 25 µM for 72 h. The polyphenol significantly increased the expression of the browning specific marker CIDE-A in a dose-dependent manner, although the classic brown adipocyte marker ZIC1 did not show any significant changes. Moreover, the authors found an elevation in the mitochondrial content and in mitochondrial biogenesis, in the PPARγ coactivator-1a (PGC-1α) marker, as well as in the expression of the mitochondrial uncoupling protein 1 (UCP1), OCR and the beige adipocyte marker TBX-1. To further investigate the mechanism behind this effect, the authors studied the implication of AMP-activated protein kinase (AMPK). Xanthohumol increased the phosphorylation of AMPK and, thus, its activation. When the phenolic compound was co-incubated with an AMPK inhibitor (dorsomorphin), the expression of UCP1 remained unchanged, and when it was co-incubated with an AMPK activator (AICAR), the expression of UCP1 was potentiated, when compared with the effect of xanthohumol alone. These results suggested that xanthohumol-induced browning is mediated by the AMPK signaling pathway. Furthermore, xanthohumol at the highest dose increased phosphorylated-acetyl-CoA carboxylase (ACC), indicating an inhibitory effect on de novo lipogenesis, and in adipose triglyceride lipase (ATGL) and hormone sensitive lipase (HSL) protein expression, indicating an increase in lipolysis.

Finally, the effects of xanthohumol have also been investigated in hepatocytes. Gao et al. (2024) studied the lipid-lowering effect of this prenylflavonoid through attenuating angiopoietin-like 3 (ANGPTL3) [27]. ANGPTL3 acts as an inhibitor of lipoprotein lipase (LPL), avoiding the breakdown of triglyceride-rich lipoproteins in circulation, such as chylomicrons and very low-density lipoproteins (VLDL). The ANGPTL family comprises eight protein members, ANGPTL3 being a promising new target for the development of therapies focused on hyperlipidemia and cardiovascular diseases [29].

By using human hepatoma Huh-7 or HepG2 cells treated with xanthohumol at 10 or 20 μM for 24 h, the authors observed, in both cell lines, a dose-dependent decrease in either ANGPTL3 mRNA expression (36% and 65%, respectively), protein expression (44% and 66%, respectively), or secreted ANGPTL3 proteins to the medium (34% and 47% lower), as compared to the control cells.

Looking for a mechanistic explanation, the authors stated that, in hepatic cells, ANGPTL3 gene expression is mainly controlled by key transcription factors, such as liver X receptor α (LXRα) and hepatic nuclear factor 1α (HNF-1α). They speculated that xanthohumol may serve as a ligand and has the potential to interact with the LXRα protein to suppress LXRα-mediated transcriptional activity in HepG2 cells. For this purpose, after checking that xanthohumol treatment did not alter the amounts of nuclear HNF-1α and/or LXRα proteins, they transfected HepG2 cells with ANGPTL3 promoter–luciferase reporter constructs including a mutated LXR-binding element (LXRE). After 24 h of transfection, the cells were pretreated with either the vehicle or xanthohumol (20 μM) for 1 h, followed by incubation with the highly selective LXR ligand T0901317 (1 μM), or co-treated with the phenolic compound (20 μM) and T0901317 (1 μM) for 24 h. As a result, cells transfected with the mutant LXRE constructs showed a significant 65% reduction in ANGPTL3 transcriptional activity compared to those transfected with the wild-type construct. Therefore, the authors concluded that LXRE is a critical responsive element for the xanthohumol-mediated reduction in ANGPTL3 transcriptional activity [27].

Miyata et al. (2015) investigated xanthohumol as a novel sterol regulatory element-binding protein (SREBP) inactivator, which is able to reduce the de novo synthesis of fatty acid and cholesterol in the liver [28]. For this purpose, the authors used a human hepatoma Huh-7 cell line that was stably expressed as a luciferase reporter gene driven by an SRE-containing fatty acid synthase (FAS) promoter (Huh-7/FAS-luc) and identified xanthohumol as a potent SREBP inactivator because it reduced the level of mature forms of SREBPs.

### Summary

The studies carried out in maturing pre-adipocytes show that xanthohumol is able to inhibit adipogenesis in a range of concentrations from 1 to 25 μM. In some cases, the effects of this phenolic compound on the two processes involved in adipogenesis, cell proliferation and cell differentiation, have been separately analyzed, thus concluding that xanthohumol reduces both of them. Concerning cell proliferation reduction, the main mechanisms underlying these effects are a decrease in Ki67 protein and an increase in apoptosis. Regarding cell differentiation, a reduction in key transcriptional factors, such as PPARγ and C/EBPα, together with a decrease in NFκB activity, seems to be involved.

In mature adipocytes, reductions in lipid accumulation have been observed after incubating these cells with xanthohumol at concentrations in a range of 3.125 to 25 μM, which is very similar to those showing anti-adipogenic effects in pre-adipocytes. This reduction in lipid accumulation can be explained by an increase in lipid mobilization and apoptosis, via increases in caspase 3/7 activity, ROS production and cytochrome c release, and decreased expression of proliferation-specific nuclear Ki67 protein. The involvement of BCL-2 and BAX proteins has not been observed in these studies. Moreover, the involvement of the white adipocyte tissue browning as another mechanism for the reduction of lipid accumulation also seems to be proved by the increase of mitochondrial biogenesis and browning master markers.

**Figure 2:**
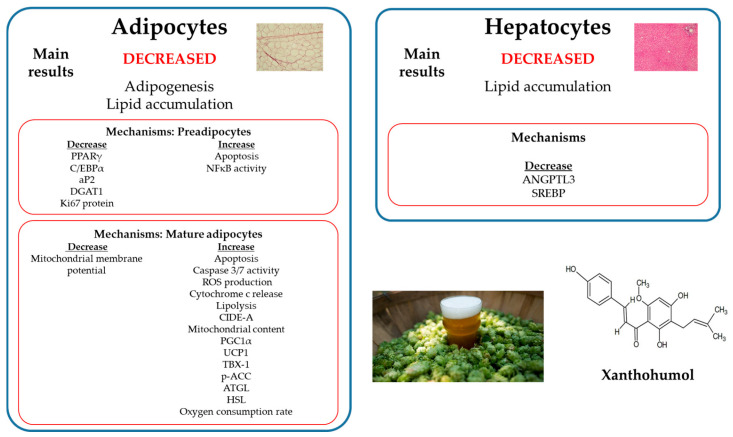
Recapitulation of the main results and the possible mechanisms involved in the effects of xanthohumol in in vitro experiments. aP2: fatty acid-binding protein 2; ATGL: adipose triglyceride lipase; C/EBPα: CCAAT/enhancer binding protein α; CIDE-A: cell death-inducing DFFA-like effector a; DGAT1: diacylglycerol O-acyltransferase 1; HSL: hormone sensitive lipase; NFκB: nuclear factor κB; p-ACC: phosphorylated acetyl-CoA carboxylase; PGC1α: peroxisome proliferator-activated receptor gamma coactivator 1α; PPARγ: peroxisome proliferator-activated receptor γ; ROS: reactive oxygen species; TBX-1: T-Box transcription factor 1; UCP1: uncoupling protein 1.

In hepatocytes, a potential pathway involved for the reduction in lipid synthesis is the inactivation of the master SBREP transcription factors involved in fatty acid and cholesterol synthesis. Further, an attenuation of angiopoietin-like 3 activity, an inhibitor of lipoprotein lipase (LPL), may be involved in the amelioration of the metabolic syndrome-associated dyslipidemia (Figure 2).

## 4. In Vivo Studies in Animal Models

Most of the animal studies have been carried out in rodents, using different diets or drugs to induce metabolic syndrome, or animals developing metabolic syndrome genetically, although other animal models (zebrafish) have also been used (Table 2). Miyata et al. (2015) studied the effect of xanthohumol on metabolic syndrome in 6-week-old male C57BL/6J diet induced obese mice [28]. For that purpose, mice were fed a high-fat diet (60% of energy from fat) for 10 weeks and, after that period, mice were maintained with the same diet for an additional 50 days, supplemented or not with 0.2% or 0.4% of the phenolic compound. Xanthohumol reduced body weight in a dose-dependent manner, as well as, liver weight, visceral fat depots (highest dose), and subcutaneous fat depot (both doses). This body weight reduction in the case of the highest dose could be attributed, in part, to a reduction in triglyceride absorption, as observed by increased triglyceride content in faeces. To better understand the potential mechanisms involved in the reduction of subcutaneous depots, different metabolic pathways were analyzed. Whereas the gene expression of de novo lipogenic enzyme acc was not modified by the treatment, fas was diminished by the lowest dose of xanthohumol. Gene expression of the lipolytic enzyme atgl was increased by both doses, but hsl remained unchanged. There were also no differences between groups in the expression of the transcription factor pparγ and protein expression of the transcription factor SREBP1. Moreover, it seems that browning could be involved in this effect because mRNA levels of ucp1 were increased in mice fed the highest dose of the phenolic compound.

In serum, triglyceride levels were not modified by the treatments, although HDL-cholesterol, LDL-cholesterol, and total cholesterol were decreased. This effect could, in part, be due to an increase in cholesterol catabolism, as suggested by the increase in the expression of hepatic genes ATP-binding cassette subfamily G member 8 (abcg8) and cholesterol 7-hydroxylase (cyp7a1). This increase was only statistically significant in the case of the highest dose. By contrast, genes involved in cholesterol synthesis, hydroxymethylglutaryl-CoA synthase (hmgcs), hydroxymethylglutaryl-CoA reductase (hmgcr), and squalene synthase (sqs), were not affected by the treatment.

With regard to insulin signaling, serum glucose tended to decrease in animals receiving xanthohumol, and insulin level was significantly lower in those animals. Hepatic proteins involved in insulin signaling were studied and an increase in protein expression of phosphorylated and total S6 kinase (S6K), as well as in phosphorylated-AMPK (not in total) were observed, indicating an activation of these proteins.

The authors also observed a lipid-lowering effect in liver. This effect seems to be due to a decrease in de novo lipogenesis (decreased mRNA levels of acc, fas and scd1), but not to an increase in fatty acid oxidation (no changes in mRNA levels acyl-CoA oxidase (aco) or carnitine palmitoyl transferase (cpt)).

Finally, due to the fact that brown adipose tissue can improve glucose and lipid metabolism, and insulin resistance, gene expression of ucp1, deiodinase, iodothyronine type 2 (dio2) and pgc1α was determined, but no differences among experimental groups were observed.

Miranda et al. (2016) carried out a study in 8-week-old male C57BL/6J mice, fed a high-fat diet (60% of energy from fat), supplemented or not with 0.033% or 0.066% of xanthohumol (estimated to be 30 mg/kg body weight/day and 60 mg/kg body weight/day, respectively), for 12 weeks [30]. At the end of the experimental period, mice treated with the phenolic compound showed lower body weight than those not supplemented. Regarding glucose homeostasis, whereas both doses decreased plasma insulin levels, only the highest one was able to avoid the increase in plasma glucose levels, thus indicating that the higher dose was more effective in reducing insulin resistance. In addition, plasma total cholesterol and LDL-cholesterol levels were reduced by both doses of xanthohumol. Only the highest dose decreased triglycerides and increased HDL-cholesterol. The reduction in LDL-cholesterol levels were probably due, at least in part, to a decrease in LDL-receptor degradation, as suggested by the decrease in plasma levels of pro-protein convertase subtilisin kexin 9 (PCSK9), a secreted protease that induces LDL-receptor degradation.

Moreover, xanthohumol was also able to reduce the pro-inflammatory state associated with obesity. In this sense, it reduced interleukin 6 (IL-6) plasma levels and, in the case of the highest dose, also reduced monocyte chemoattractant protein 1 (MCP-1) levels, a marker of macrophage infiltration. Plasma leptin, an adipokine involved in food intake and body weight regulation, was decreased by the phenolic compound (both doses), suggesting a decrease in leptin resistance. In summary, in mice showing diet-induced obesity, xanthohumol prevented body weight gain and ameliorated dyslipidemia, hyperglycemia, insulin resistance, and fatty liver. Moreover, it decreased the pro-inflammatory state related to obesity and insulin resistance.

Deepening the study of the amelioration of the lipid metabolism by xanthohumol, Hirata et al. (2017) studied the effects of xanthohumol on reverse cholesterol transport (RCT) and HDL-cholesterol levels in an in vivo hamster model [31]. The effects of 0.1% of xanthohumol through a high-cholesterol diet, for 6 weeks, showed that the intake of this compound enhanced reverse cholesterol transport through cholesterol efflux from macrophages and excretion to faeces.

Later on, Miranda et al. (2018) evaluated the effect of 30 mg/kg body weight/day of xanthohumol on male C57BL/6J mice fed a high-fat diet (60% of total calories from fat) for 13 weeks [32]. At the end of the treatment, changes neither in body weight gain nor in food intake were observed, when compared to the control group. Additionally, fasting triglyceride, cholesterol, and leptin plasma concentrations remained unchanged. Treatment of mice with this compound did not cause an increase in plasma AST levels, while the ALT was significantly reduced, which may indicate a xanthohumol-induced hepatoprotective effect. These results are not in good accordance with those previously observed by the same group [30]. The authors explained that in the previous study mice were 8-week-old and showed an initial body weight of 21.6 ± 0.03 g, whereas in the present study mice were 9-week-old and showed a body weight 24.1 ± 0.25 g. They considered that these differences might explain the difference in body weight gain over time because fatness has been shown to be the strongest predictor of the variability in weight gain independent of the duration of HFD feeding. Plasma glucose and insulin levels, and thus HOMA-IR index, were not improved by the treatment. Finally, xanthohumol treatment increased the activation of AMPK in the liver and inhibited AMPK activation in skeletal muscle, but the authors considered that these effects were probably not directly related to the improvement in glucose metabolism.

In an experiment reported by Mahli et al. (2019), male C57BL/6 mice were fed either a standard diet or a western-diet (a high-fat, high-sucrose diet) for 3 weeks [33]. After that period, mice fed the western-diet continued with this diet and received or not 2.5 mg of xanthohumol/kg body weight/day by oral gavage, for 8 additional weeks. Xanthohumol was administered either on its native form or after its micellar solubilisation in order to increase its oral bioavailability. Mean serum xanthohumol concentrations were 166 nmol/L in mice treated with the solubilized form, whereas it was not detectable when it was administered on its native form, showing an improvement on its bioavailability.

Xanthohumol treatment reduced body weight, compared to the non-treated animals fed the western diet, but the reduction was significant only with the micellar preparation. The weight of the animals did not reach the values of the control animals. Regarding white adipose tissue, the solubilised form of xanthohumol significantly prevented the increase in visceral and subcutaneous depots. Moreover, xanthohumol after micellar solubilisation, but not on its native form, significantly reduced both fasting glucose levels and glucose levels when the glucose tolerance test was carried out. In addition, only as micellar solubilised xanthohumol was able to reduce hepatic index (liver weight/body weight) and hepatic triglyceride content. Similarly, lower hepatic mRNA levels of mcp-1 and chemokine (C-X-C motif) ligand 1 (cxcl1), two markers of inflammation, were found when xanthohumol was solubilised in comparison with animals fed the western diet without supplementation. These results were confirmed by CD3 immunohistochemical staining. Regarding fibrosis, the solubilised form of xanthohumol also reduced gene expression of α-smooth muscle actin (αsma) and collagen-α-1, as well as the protein quantity of αSMA. Based on these results, the authors concluded that xanthohumol is a promising agent for the improvement of different components of the metabolic syndrome including obesity, insulin resistance, and fatty liver, and that its biological efficacy improved with the micellar solubilization.

Wang et al. (2021) investigated the effects of xanthohumol on liver steatosis and fibrosis that were induced by the presence of type 2 diabetes, which was provoked by the intraperitoneal administration of a single dose of streptozotocin [34]. Diabetic male Sprague Dawley rats fed a standard diet, or a high-fat diet supplemented or not with a low (5.64 mg/kg body weight/day), or a high (16.9 mg/kg body weight/day) dose of xanthohumol from the third week of the experiment to 12th week. This phenolic compound ameliorated the induced alterations in fasting glucose and insulin levels, triglycerides, total cholesterol, HDL-cholesterol, LDL-cholesterol, VLDL-cholesterol, as well as ALT and AST levels in a dose-dependent manner. Furthermore, it also improved HOMA-IR, HOMA-B, and QUICKI indexes. Moreover, serum inflammatory (IL-1β, IL-6, and TNFα) markers were significantly lower in rats treated with the phenolic compound, indicating a lower inflammatory status. As far as oxidative stress is concerned, serum superoxide dismutase (SOD) and reduced glutathione (GSH) levels were higher in rats fed xanthohumol and lower for malonaldehyde (MDA), suggesting a better control of oxidative stress.

Additionally, hepatic histological analysis revealed that xanthohumol effectively reduced triglyceride accumulation and inflammation. Concerning fibrosis, the phenolic compound also decreased the protein expression of αSMA, TGF-β1, SMAD3, and COL1A2, indicating an improvement in hepatic fibrosis associated with the NAFLD.

Logan et al. (2021) hypothesized that this phenolic compound requires the microbiota to exert its beneficial effects [35]. To test this hypothesis, conventional and germ free male Swiss Webster mice were fed with either a low-fat diet, a high-fat diet, or a high-fat diet supplemented with xanthohumol at 60 mg/kg body weight/day for 10 weeks. The supplementation with this compound did not decrease final body weight or body weight gain. Plasma lipid (cholesterol profile) and glucose alteration associated with the high-fat diet also remained unchanged. Surprisingly, xanthohumol increased plasma triglycerides in germ free mice, but not in conventional mice. On the other hand, this compound significantly decreased insulin levels and HOMA-IR in conventional mice, but not in germ free mice. This result indicates that xanthohumol is only effective in the presence of intestinal microbiota. Regarding muscular and hepatic triglycerides and cholesterol, no changes were observed when compared to mice fed the same high-fat diet without the phenolic compound.

Further, the authors analyzed how the diet affected the taxonomic composition of the gut microbiome and, interestingly, it was found that supplementation with xanthohumol decreased both α- and β-diversity, but not β-dispersion. Compared to the high-fat diet, xanthohumol treatment enhanced the relative abundance of *Verrucomicrobiaceae* family and reduced the relative abundance of *Porphyromonadaceae*, *Lactobacillaceae*, and *Lachnospiraceae* families. Analysis of amplicon sequence variants (ASVs) revealed that xanthohumol supplementation up-regulated the relative abundance of *Akkermansia muciniphila*, *Parabacteroides goldsteinii*, *Alistipes finegoldii*, and an unknown species of the Bacteroides genus. Thus, administration of xanthohumol changed the predicted microbial functional capacity, and with more impact than the high-fat diet.

In addition to diet- or drug-induced metabolic syndrome models, genetic animal models have been used to study the effects of xanthohumol (Table 2). Legette et al. (2013) carried out experiments in a genetic model of obesity (Zucker *fa/fa* rats) [36]. Four-week-old male and female obese Zucker rats were fed a high-fat diet (60% of energy from fat) for 3 weeks to induce severe obesity; subsequently rats were switched to a standard diet for 3 additional weeks. At the end of the experimental period, rats were distributed into three experimental groups: one group was fed only the standard diet, and the other two groups were supplemented with xanthohumol at 1.86 mg/kg body weight/day, 5.64 mg/kg body weight/day or 16.9 mg/kg body weight/day. The phenolic compound was administered mixed with the diet. Reductions in body weight and plasma glucose levels were observed only when the male rats received the highest dose of xanthohumol, suggesting that this compound can be effective in obesity for glucose homeostasis.

Takahashi et al. (2017) studied the effect of various bioactive compounds in a genetic mice model of obesity and type 2 diabetes [37]. Four-week-old KK-Ay/TaJc1 mice were fed a standard diet supplemented or not with 0.2% of xanthohumol for 81 days (around 286 mg/kg/d). Although no differences in body weight were observed, the mice supplemented with the phenolic compound showed a significant reduction in adipose tissue weight (epidydimal, perirenal, and mesenteric) when compared to the control mice. No changes in plasma triglyceride, free fatty acids, total cholesterol, HDL-cholesterol, blood glucose, glycosylated hemoglobin A1c (HbA1c), insulin, and adiponectin levels were observed in xanthohumol-treated mice. By contrast, plasma leptin levels were lower in mice fed the phenolic compound.

With regard to liver parameters, whereas no differences in liver weight or hepatic triglyceride content were found between both experimental groups, mice treated with xanthohumol showed lower hepatic cholesterol content. At a molecular level, xanthohumol reduced gene expression of srebp1c, fas and malic enzyme, although statistical significance was only reached in the case of Srebp1c. Regarding the genes related to fatty acid oxidation, an increase in pparα, aco and cpt2 was observed, suggesting and increase in hepatic fatty acid oxidation.

Paraiso et al. (2021) evaluated the effects of xanthohumol on liver damage induced by diet and farnesoid X receptor (FXR) deficiency [38]. For that purpose, 10-week-old male and female wild type mice and a liver-specific FXR-null (FXR Liver −/−) C57BL/6J mice were fed a high-fat diet with the phenolic compound (60 mg/kg body weight/day mixed with the diet) or the vehicle. At the end of the experimental period 812 weeks), no changes in body weight or plasma glucose and ALT were observed when mice were treated with xanthohumol. However, the phenolic compound significantly reduced plasma AST levels in FXR Liver−/− mice. Regarding liver composition, stained sections showed a delipidating effect induced by xanthohumol in both genotypes of mice. This effect was higher in males than in females. These results indicate that xanthohumol regulates lipid metabolism via pathways independent of hepatic FXR signaling.

Finally, the lipid-lowering effect of xanthohumol has been also investigated by using zebrafish as an animal model [27]. Adult zebrafish were divided into two groups and fed a normal diet (ND) or a high-fat diet (HFD), consisting in the normal diet enriched with lard oil (20%), for six weeks. After four weeks of the experimental period, the HFD-fed group was further divided into two subgroups. One of these subgroups received xanthohumol (10 mg/kg of body weight); the other group, along with the animals in ND group, received a vehicle (DMSO) each two days for the remaining 14 days. At the end of the experimental period, body weight was measured, and blood and liver were obtained.

Whereas HFD led to an increase in the final body weight of the animals, no differences were observed in this parameter between the animals offered the HFD supplemented or not with xanthohumol. However, when the authors assessed plasma lipid levels in the zebrafish animal models, xanthohumol decreased the levels of plasma triglycerides and cholesterol compared to those in the HFD (vehicle) group, suggesting that xanthohumol can improve the HFD-induced dyslipidemia. Further, as previously explained in this review, these authors were interested in investigating the impact of xanthohumol on hepatic ANGPTL3 levels, which acts as an inhibitor of lipoprotein lipase (LPL). HFD feeding promoted a substantial increase (2.1-fold higher) in hepatic ANGPTL3 protein levels as compared to those in the ND (vehicle) group. When xanthohumol was provided to the HFD-receiving animals, a significant reduction (−42%) in hepatic ANGPTL3 protein levels was observed. Further, LPL activity was significantly increased in the protein lysates from this group of animals compared to the HFD (vehicle) group. This suggests that xanthohumol supplementation inhibited hepatic ANGPTL3 expression in the HFD fed animals, leading to an increased LPL activity. This, in combination with the observed reduction of plasma triglycerides and cholesterol levels, prompted the authors to conclude that xanthohumol serves to ameliorate dyslipidemia in vivo.

### Summary

When the experimental designs used in the studies addressed in rodent models are compared, important differences are observed. Thus, the doses range from 1.86 mg/kg body weight/day to 286 mg/kg body weight/day, and the experimental period ranges from 6 to 13 weeks. In addition, the age of animals was quite different, ranging from 4 to 11 weeks.

In general terms, it can be said that xanthohumol is able to improve NAFLD. This delipidating effect is due, at least in part, to a reduction in de novo lipogenesis, and probably to better glucose control. Moreover, this phenolic compound seems to reduce white adipose depots and thus obesity. This effect can be mediated by an increase in lipolysis and to a browning effect on white adipose tissue. Most of studies revealed an improvement in dyslipidemia.

**Figure 3:**
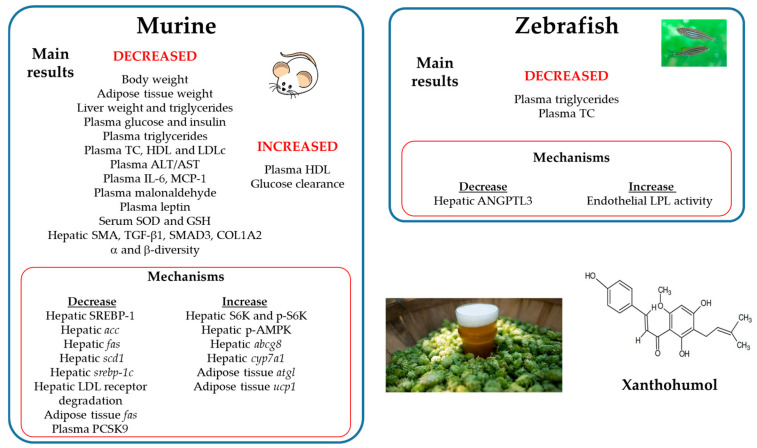
Recapitulation of the main results and the possible mechanisms involved in the effects of xanthohumol in in vivo experiments. *abcg8*: ATP-binding cassette subfamily G member 8; ALT: alanine aminotransferase; AMPK: AMP-activated protein kinase; ANGPTL3: angiopoietin-like 3; AST: aspartate aminotransferase; COL1A2: collagen 1A2; *cyp7a1*: cholesterol 7-hydroxylase; GSH: reduced glutathione; HDL: high density lipoprotein; IL-6: interleukin-6; LDL: low density lipoprotein; LPL: lipoprotein lipase; MCP-1: monocyte chemoattractant protein 1; *scd*-1: stearoyl CoA desaturase-1; SMA: smooth muscle actin; SMAD3: small mother against decapentaplegic 3; SREBP: sterol regulatory element-binding protein; S6K: S6 kinase; SOD: superoxide dismutase; TC: total cholesterol; TGF-β1: Transforming growth factor-β1; *ucp1*: uncoupling protein 1.

Nevertheless, several differences exist among the reported studies in the observed results. When looking at the experimental designs, in order to find reasons that could explain controversial results, no clear conclusions are drawn. Thus, whereas in the study published by Takahashi et al. [37] no changes in body weight, plasma triglycerides, free fatty acids, total cholesterol, HDL-cholesterol, glucose, insulin, and hepatic triglycerides were found using 286 mg/kg body weight/day (the highest dose used in all the reported studies); other studies have found changes in these parameters using doses clearly lower. This discrepancy could be related to the animal model used, male KK-Ay/TaJc1 mice fed a standard diet in the study reported by Takahashi et al. [37], instead of C57BL/6J mice fed a high-fat diet in other studies. Nevertheless, it is important to point out that even in studies where similar species and strains of animals were used, discrepancies in the results are obtained. Thus, Miranda et al. [32] observed clear improvement in biochemical serum parameters in a study carried out in C57BL/6J mice fed a high-fat diet, but not in another experiment performed later on with the same type of mice. As described previously in this review, the authors attributed this discrepancy to differences in the age and initial body weight of mice. Finally, whereas Legette et al. [36], using doses of 1.86, 5.64, and 16.9 mg/kg body weight/day, only observed significant effects with the highest dose, Wang et al. [34] found significant effects with the dose of 5.64 mg/kg body weight/day. No clear reasons to explain this discrepancy are found (Figure 3).

## 5. Conclusions

The in vitro and in vivo studies gathered in the present review suggest that xanthohumol is a polyphenol with potential applications in obesity because it is able to reduce body weight and adipose tissue weights in animal models, as well as to improve some metabolic alterations related to this pathology, such as dyslipidaemias, insulin resistance, and fatty liver. Nevertheless, several drawbacks exist, and thus further investigation is needed. More studies should be addressed to establish a range of effective doses and to better characterize the mechanisms of action underlying the beneficial effects induced by this polyphenol. As can be seen in Table 2, only a reduced number of studies has focused on this issue. On the other hand, all the studies reported so far have been addressed in rodent models. Thus, clinical intervention studies are needed to check whether the interesting effects found in animals are reproduced in human beings.

Finally, taking into account that the only relevant source of xanthohumol is beer, and that the amount of this polyphenol in this drink is low, as described in the Section 1 of this review, probably the best way to enjoy the health benefits of xanthohumol is by using it as a nutraceutical in functional foods. Food supplements providing extracts with xanthohumol dosages ranging between 20 and 50 mg are found on the market (SuperSmart^TM^: www.super-smart.eu; Altruvita^TM^: www.altruvita.com (accessed 7 November 2024)).

## Figures and Tables

**Table 1:** In vitro effects of xanthohumol on adipocytes and hepatocytes.

Reference	Experimental Design	Main Results	Mechanism of Action
[23]	3T3-L1 maturing adipocytes Xanthohumol: 3.125, 6.25, 12.5, or 25 μM 6 days during adipogenesis 3T3-L1 mature adipocytes Xanthohumol: 25, 50, 75, or 100 μM 24 or 48 h	↓ Lipid accumulation in maturing adipocytes (all doses) ↓ Cell viability in mature adipocytes (50–100 μM)	On adipogenesis: ↓ PPARγ, C/EBPα, aP2, and DGAT1 On mature adipocytes: ↑ apoptosis, caspase 3/7 activity, ROS production and cytochrome c release; ↓ mitochondrial membrane potential
[24]	3T3-L1 maturing adipocytes Xanthohumol: 1.5 or 3.12 μM 6 days during adipogenesis 3T3-L1 mature adipocytes Xanthohumol: 12.5 or 25 μM 24 or 48 h	↓ Lipid accumulation in maturing adipocytes (both doses) ↓ Cell viability in mature adipocytes (25 μM)	On adipogenesis: unknown On mature adipocytes: ↑ caspase 3/7 activity and lipolysis
[25]	3T3-L1 maturing adipocytes Xanthohumol: 0.1, 1, 10, or 20 μM During adipogenesis: From day −2 to day 7 From day −2 to day 0 From day 0 to day 7 From day 0 to day 2 From day 2 to day 7)	↓ Lipid accumulation in maturing adipocytes 1 μM all the conditions except from day −2 to day 0 and from day 2 to 7 10 and 20 μM all the conditions except from day 2 to day 7 ↓ Cell proliferation	Preadipocytes: ↓ Ki67 protein; ↑ apoptosis, NFκB activity Adipocytes: ↑ apoptosis
[26]	3T3-L1 and primary human subcutaneous maturing adipocytes Xanthohumol: 6.25 or 25 μM 6 days during adipogenesis respectively 3T3-L1 mature adipocytes Xanthohumol: 25 μM 72 h	↓ Lipid accumulation in maturing adipocytes (high dose in both cell lines) ↓ Lipid accumulation in mature adipocytes	On adipogenesis: unknown On mature adipocytes: ↑ CIDE-A (both doses), mitochondrial content, PGC1α, UCP1, TBX-1, p-ACC, ATGL, HSL protein expression, and oxygen consumption rate
[27]	HepG2 and Huh-7 hepatocytes Xanthohumol: 10 or 20 μM 24 h	↓ secreted ANGPTL3	↓ LXRα activity (high dose)
[28]	Huh-7 hepatocytes Xanthohumol: 10 or 20 μM 24 h	↓ de novo synthesis of fatty acid and cholesterol	↓ SREBP activity ↑ *Acc* gene expression (low dose) ↓ *Fas, scd1, hmgcs, hmgcr*, and *sqs* gene expressions (both doses)

ACC: acetyl CoA carboxylase; ANGPTL3: angiopoietin-like 3; aP2: fatty acid-binding protein 2; ATGL: adipose triglyceride lipase; C/EBPα: CCAAT/enhancer binding protein α; CIDE-A: cell death-inducing DFFA-like effector a; DGAT1: diacylglycerol O-acyltransferase 1; *Fas*: fatty acid synthase; *hmgcr*: 3-hydroxy-3-methyl-glutaryl-coenzyme A reductase; *hmgcs*: 3-hydroxy-3-methyl-glutaryl-coenzyme A synthase; HSL: hormone sensitive lipase; LXRα: liver X receptor α; NFκB: nuclear factor κB; p-ACC: phosphorylated acetyl-CoA carboxylase; PGC1α: peroxisome proliferator-activated receptor gamma coactivator 1α; PPARγ: peroxisome proliferator-activated receptor γ; ROS: reactive oxygen species; *scd1*: stearoyl-CoA desaturase 1; *sqs*: squalene synthase; SREBP: sterol regulatory element-binding protein; TBX-1: T-Box transcription factor 1; UCP1: uncoupling protein 1; ↑: increase; ↓: decrease.

**Table 2:** In vivo effects of xanthohumol on metabolic syndrome components.

Reference	Experimental Design	Xanthohumol Administration	Main Results
[28]	Animals: 6-week-old male C57BL/6J mice Diet: 10 weeks with a high-fat diet (60% of energy from fat) + 50 days with the same diet supplemented or not with xanthohumol Experimental period: 50 days n = 5 animals/group	Mixed with the diet: 0.2% 0.4%	↓ Body weight (both doses) ↓ White adipose tissue weight: subcutaneous (both doses), epididymal, mesenteric and inguinal (high dose) NS Serum triglycerides and glucose ↓ Serum LDL, HDL and total cholesterol and insulin (both doses ↑ Triglycerides in faeces (highest dose) ↓ Liver weight and fat Potential mechanisms: ↑ Hepatic protein expression of phosphorylated and total S6K and phosphorylated AMPK (both doses) ↓ Hepatic protein expression of SREBP-1 (both doses) NS Hepatic mRNA levels of *HMGCS*, *HMGCR*, *SQS*, *SREBP-2*, *ABCA1, ABCG5*, *PPARα*, *ACO*, *CPT-1a* ↓ Hepatic mRNA levels of *ACC*, *FAS*, *SCD1*, *SREBP-1c* (both doses) ↑ Hepatic mRNA levels of *ABCG8*, *CYP7A1* (highest dose) NS subcutaneous protein expression of SREBP-1 NS subcutaneous mRNA levels of *ACC*, *HSL*, *PPARγ* ↓ Subcutaneous mRNA levels of *FAS* (lowest dose) ↑ Subcutaneous mRNA levels of *ATGL* (both doses) and *UCP1* (highest dose) NS Brown adipose tissue mRNA levels of *UCP1*, *DIO2*, *PGC1α*
[30]	Animals: 8-week-old male C57BL/6J mice Diet: high-fat diet (60% of energy from fat) Experimental period: 12 weeks n = 16 animals/group s	Mixed with the diet: 0.033% (estimated 30 mg/kg body weight/day) 0.066% (estimated 60 mg/kg body weight/day)	↓ Body weight (both doses) ↓ Plasma glucose (highest dose) and insulin (both doses) ↓ Plasma triglycerides (highest dose) ↓ Plasma total cholesterol and LDL-cholesterol (both doses) ↑ Plasma HDL-cholesterol (highest dose) ↓ Hepatic triglycerides (both doses) ↓ Plasma IL-6, MCP-1 ↓ Plasma leptin **Potential mechanisms:** ↓ LDL-receptor degradation ↓ Pro-protein convertase subtilisin kexin 9 (PCSK9)
[31]	Animals: 6-week-old male Syrian hamsters Diet: mild-fat diet containing 0.5% (wt/wt) cholesterol (high-cholesterol diet; HCD) Experimental period: 6 weeks n = 12 animals/group s	Mixed with the diet: 0.1% of xanthohumol	NS Body weight ↓ Plasma total cholesterol NS Plasma LDL and HDL cholesterol
[32]	Animals: 9-week-old male C57BL/6J mice Diet: high-fat diet (60% of energy from fat) Experimental period: 13 weeks n = 5 animals/group	Mixed with the diet: 30 mg/kg body weight/day	NS Body weight NS Fasting plasma glucose, insulin, triglycerides, total cholesterol and AST ↓ Plasma ALT NS HOMA-IR ↑ Glucose clearance (4 weeks of treatment)
[33]	Animals: 8-week-old male C57BL/6 mice Diet: high-fat high-sucrose diet Experimental period: 11 weeks n = 6 animals/group	Gavage for the last 8 weeks (native or micellar xanthohumol): 2.5 mg of xanthohumol/kg of body weight/day	↓ Body weight and white adipose tissue (micellar xantho-solubilisate) ↓ Fasting serum glucose (micellar xantho-solubilisate) ↓ Hepatic triglycerides (micellar xantho-solubilisate)
[34]	Animals: diabetic male Sprague-Dawley rats (streptozotocin) Diet: high-fat diet Experimental period: 12 weeks n = 10 animals/group	Mixed with the diet from 3rd to 12th week: 5.64 mg/kg body weight/day 16.9 mg/kg body weight/day	Both doses: NS Body weight ↓ Fasting serum glucose, insulin, triglycerides, LDL-cholesterol, VLDL-cholesterol, total cholesterol, AST and ALT ↑ Serum HDL-cholesterol levels ↓ HOMA-IR, HOMA-β, QUICKI ↑ Serum SOD, GSH ↓ MDA ↓ Hepatic lipids and inflammation ↓ Hepatic protein expression of α-SMA, TGF-β1, SMAD3, COL1A2
[35]	Animals: 9- to 11-week-old male Swiss Webster (convectional and germ free) Diet: high-fat diet Experimental period: 10 weeks n = 8–10 animals/group	Mixed with the diet: 60 mg/kg body weight/day	NS Body weight NS Plasma HDL-cholesterol, LDL-cholesterol, glucose ↑ Plasma triglycerides (in germ free mice) ↓ Insulin ↓ HOMA-IR NS hepatic triglycerides and cholesterol **Potential mechanisms:** ↓ alpha-diversity ↓ beta-diversity ↑ *Verrucomicrobiaceae*, *Akkermansia muciniphila*, *Parabacteroides goldsteinii*, *Alistipes finegoldii* ↓ *Porphyromonadaceae*, *Lactobacillaceae*, and *Lachnospiraceae families*.
[36]	Animals: 4-week-old male and female obese *fa/fa* Zucker rats Diet: 3 weeks with a high-fat diet (60% of energy from fat) + 3 weeks with a standard diet Experimental period: 6 weeks n = 6 animals/group	Mixed with the diet: 1.86 mg/kg body weight/day 5.64 mg/kg body weight/day 16.9 mg/kg body weight/day	↓ Body weight (highest dose in male rats) ↓ Plasma glucose (highest dose in male rats)
[37]	Animals: 4-week-old male KK-Ay/TaJc1 mice Diet: standard diet Experimental period: 81 days n = 6–7 animals/group	Mixed with the diet: 0.6% (estimated 286 mg/kg body weight/day)	NS Body weight ↓ White adipose tissue weight NS Plasma triglycerides, free fatty acids, total cholesterol, HDL-cholesterol, glucose, HbA1c and insulin NS Hepatic triglycerides
[38]	Animals: 10-week-old male and female wild type and FXR Liver−/− C57BL/6J mice Diet: high-fat diet (60% of energy from fat) Experimental period: 12 weeks n = 7–10 animals/group	Mixed with the diet 60 mg/kg body weight/day	NS Body weight NS Plasma glucose and ALT ↓ Plasma AST (FXR Liver−/− mice) ↓ Hepatic lipids (both genotypes)
[27]	Animals: adult zebrafish Diet: high-fat diet Experimental period: 6 weeks n = 13–20 animals/group	Gavage for the last 2 weeks: 10 mg of xanthohumol/kg of body weight each two days	NS Body weight ↓ plasma triglycerides and cholesterol **Potential mechanisms:** ↓ hepatic protein expression of ANGPTL3 ↑ LPL activity

*ABCG8*: ATP-binding cassette subfamily G member 8; ALT: alanine aminotransferase; AMPK: AMP-activated protein kinase; ANGPTL3: angiopoietin-like 3; AST: aspartate aminotransferase; COL1A2: collagen 1A2; FXR: farnesoid X receptor; GSH: reduced glutathione; HbA1c: glycosylated hemoglobin A1c; HDL: high density lipoprotein; *HMGCR*: hydroxymethylglutaryl-CoA reductase; *HMGCS*: hydroxymethylglutaryl-CoA synthase; HOMA-IR: homeostatic model assessment for insulin resistance; HOMA-β: HOMA of beta cell function; IL-6: interleukin-6; LDL: low density lipoprotein; LPL: lipoprotein lipase; MCP-1: monocyte chemoattractant protein 1; n: sample size; NS: no significant changes; QUICKI: quantitative insulin sensitivity check index; *SCD1*: stearoyl CoA desaturase-1; SMAD3: small mother against decapentaplegic 3; *SQS*: squalene synthase; SREBP: sterol regulatory element-binding protein; S6K: S6 kinase; SOD: superoxide dismutase; TGF-β1: Transforming growth factor-β1; *UCP1*: uncoupling protein 1. ↑: increase; ↓: decrease.
